# Resveratrol Attenuates Aortic Dissection by Increasing Endothelial Barrier Function Through the SIRT1 Pathway

**DOI:** 10.1097/FJC.0000000000000837

**Published:** 2020-04-22

**Authors:** Kaijie Wang, Jinping Zhao, Wenwen Zhang, Minglin Zhu, Ming Xu, Dan Li, Hongjie Shi, Ming Zhang, Jiajun Shi, Zhe Dong

**Affiliations:** * Department of Thoracic and Cardiovascular Surgery, Zhongnan Hospital of Wuhan University, Wuhan, China; and; † Department of Burn and Plastic Surgery, Huaihai Hospital Affiliated to Xuzhou Medical University, Xuzhou, China.

**Keywords:** resveratrol, aortic dissection, sirtuin 1, endothelial cells, inflammatory cells

## Abstract

Aortic dissection (AD) is a serious condition and a health issue on a global scale. β-Aminopropionitrile–induced AD in mice is similar to the pathogenesis of AD in humans. Resveratrol (RSV) is a natural polyphenolic substance that provides anti-inflammatory and cardiovascular effects, but the role of RSV in AD is unclear. In this study, we investigated the effects and mechanisms of RSV on β-aminopropionitrile–induced AD in mice. Our results indicate that RSV can prevent the occurrence of AD. More meaningfully, we found that the protective effect comprises an increase in sirtuin 1 (SIRT1) expression in endothelial cells for the reconstruction of their structure, reducing the recruitment of inflammatory cells by endothelial cells and inhibiting the inflammation response, thereby suppressing the occurrence of AD.

## INTRODUCTION

Aortic dissection (AD) is a cardiovascular disease that threatens humans, with a rate of high mortality and disability. The aortic wall is composed of the following 3 layers: intima, middle layer, and adventitia. AD involves the rupture of the aortic intimal layer because of either internal or external causes. As a result, blood flows into the middle layer of the aortic wall through the rupture, causing the intimal and adventitia to separate, forming a true cavity and a false cavity.^1^ Without a timely and effective treatment, and as the disease progresses, the aortic wall will rupture, which can result in severe hemorrhaging. According to the DeBakey classification, AD is classified into type I, type II, and type III. Types I and II involve the ascending aorta, such that these types are more dangerous, wherein the probability of AD is high.^2^ Many patients with types I and II of AD die before treatment. Moreover, in recent decades, the incidence of hypertension and atherosclerotic diseases has gradually increased both of which are considered risk factors of AD.^3^ This has resulted in an upward trend in the incidence of AD, which has led to an increase in research focused on the etiology of AD.

Historically, researchers have believed that the inflammatory response does not participate in the degradation of the middle aortic layer.^4^ However, research is increasingly showing that the inflammation mechanism participates in the degradation of the middle aortic layer and plays a key role in the formation of the AD. Studies have found that macrophages accumulate in the aortic wall when AD occurs. These cells not only increase protease expression but also contribute to aortic smooth muscle cell apoptosis, which leads to the degradation of the middle aortic layer.^5^ More importantly, multiple genes encoding for proinflammatory factors have been found to be expressed in patients with AD.^6^ This evidence suggests that when AD occurs, inflammatory mechanisms may be involved in aortic wall structural remodeling.

As intimal rupture is one of the initial events of AD, changes in the intimal structure result in changes to endothelial cell function.^7^ Increased vascular endothelial permeability is one of the most important features of endothelial cell barrier dysfunction. In this process, vascular endothelial inflammation provides an important area for innate immune cells.^8^ Jia et al^9^ found that when AD occurs in mice, the vascular endothelium undergoes an inflammatory response, leading to vascular endothelial dysfunction. Other studies also have shown that when the connection between vascular endothelial cells is damaged, the permeability of endothelial cells increases and immune cells penetrate into the vascular wall through endothelial cells, destroying the integrity of the vascular wall, thereby promoting the formation of AD.^10,11^ The associated protein changes in the cytoskeleton and during cell migration play key roles in cell permeability.^12^ However, the specific mechanism remains unclear. Therefore, we hypothesize that endothelial cell dysfunction plays a role in the formation of AD.

Resveratrol (RSV) is a natural polyphenolic substance found in plants, including grapes and peanuts. Its many beneficial effects, including antiaging, anticancer, anti-inflammatory, and prevention of cardiovascular diseases, have attracted researchers' attention.^13^ The vasoprotective mechanism of RSV involves reducing oxidative stress and inhibiting inflammatory responses.^14^ Research shows that RSV can slow the expansion rate of abdominal aortic aneurysms.^15^ Aneurysms and dissections are closely related. The enlargement of aneurysms can lead to the development of ADs. Similarly, dissections can also appear as aneurysms, and the pathological changes of the 2 are similar. For example, the degradation of the middle aortic layer plays an important role in the pathogenesis of both.^16^

To the best of our knowledge, the role of RSV in AD has not yet been studied. This study aims to explore the role and mechanism of RSV in AD by using RSV to treat β-aminopropionitrile (BAPN)-induced AD in mice.

## MATERIALS AND METHODS

### Animal Experiment

Eighty male C57BL/6J mice were purchased from the Animal Experiment Center of Wuhan University. They were randomly divided into 4 groups as follows: Vehicle group, AD + Vehicle group, AD + RSV group, and AD + RSV + EX-527 group. Mice received oral BAPN (0.25 mg/kg/d, Solarbio, Beijing, China) to induce the AD. In the AD + RSV group and the AD + RSV + EX-527 group, RSV [vehicle: dimethyl sulfoxide (DMSO), 5 mg/kg/d, SC0276, Beyotime, Shanghai, China] or oral SIRT1-specific inhibitor EX-527 (10 mg/kg/d, Hy-15452, MCE) was injected intraperitoneally 1 week before the induction of AD. In addition to oral BAPN, mice in the AD + Vehicle group were injected intraperitoneally with the same amount of DMSO (D5879; Sigma) 1 week before the induction of the AD. Mice in the Vehicle group were administered the same amount of DMSO intraperitoneally. The entire animal experiment cycle was for 5 weeks. The changes in the weight of the mice and the number of ADs were recorded. After 5 weeks, the mice were killed using excess tribromoethanol (Aladdin, Shanghai, China). The aorta of the mice was subsequently isolated and placed in 4% paraformaldehyde and liquid nitrogen. All animal experiments were approved by the Ethics Committee of the Animal Experiment Center of Wuhan University.

### Aortic Histology

The aorta fixed in 4% paraformaldehyde was embedded in paraffin and cut into sections with a thickness of 5 µm. The structural changes of the aortic wall were observed by hematoxylin and eosin and Verhoeff–Van Gieson staining.

### Aortic Immunohistochemistry

The 4% paraformaldehyde-fixed aorta was embedded in paraffin and cut to a thickness of 5 µm. The tissues were blocked in bovine serum albumin (BIOFROXX, Ludwigsfelde-Genshagen, Germany) for 30 minutes before being incubated with primary antibodies CD68+ (1:100, ab125212; Abcam) at 4°C overnight. The tissues were then incubated with the goat anti-mouse (1:100,074-1506; KPL) antibody for 60 minutes. The positive cells were brown. CD68+ was used to mark the macrophages. Ten areas of each sample were randomly selected. The number and area of positive cells were analyzed using Image-Pro Plus (Version 6.0). Average optical density was used to determine the amount of positive cell expression.

### Cell Culture and Treatment

Human umbilical vein endothelial cells (HUVECs; Sciencell, San Diego, CA) were cultured in an endothelial cell medium (Catalog #1001; Sciencell) containing 5% fetal bovine serum, 1% penicillin/streptomycin, and growth factors. THP-1 macrophages (ATCC; ATCC TIB‐202, Manassas, VA) were cultured in an RPMI 1640 medium (SH30809.01; GE, Healthcare Life Sciences, Logan, UT) containing 10% inactivated fetal bovine serum, 1% penicillin/streptomycin, and 0.05-mM β-mercaptoethanol (Sigma, Merck KgaA, Darmstadt,Germany). Cells were incubated in 5% CO_2_ at 37°C before digesting with 0.25% trypsin. The mixture was centrifuged at 1000 rpm for 5 minutes. The resulting cell pellet was resuspended in an endothelial cell medium or an RPMI medium, before being seeded into culture flasks. According to the purpose of the experiment, the cells were divided into a Vehicle group, RSV group, Vehicle + Lipopolysaccharide (LPS) group, RSV + LPS group, and RSV + LPS + EX-527 group. The RSV group was added 10 µM RSV, whereas the Vehicle group was added the same amount of vehicle. The Vehicle + LPS group was induced with 500 ng/mL of LPS and an equivalent amount of vehicle for 24 hours. The RSV + LPS and RSV + LPS + EX-527 groups were incubated with RSV (10 µM) or EX-527 (10 µM) overnight before inducing HUVECs with LPS.

### Macrophage and Endothelial Cell Adhesion

Carboxyfluorescein diacetate succinimidyl ester (C1031; Beyotime, Shanghai, China) was used to label THP-1 macrophages. After the above endothelial cells were treated, the labeled THP-1 macrophages were added to each group of endothelial cells. After 4 hours, the endothelial cell supernatant was removed, and the macrophage adhesion was observed under a fluorescent inverted microscope (IX51, OLYMPUS, Tokyo, Japan).

### Cellular Immunofluorescence

According to the cell grouping, after the above treatment, the cells are fixed in 4% paraformaldehyde. The membrane was broken according to the target protein with the membrane-breaking solution at room temperature for 10 minutes. The cells were then washed with phosphate-buffered saline and added with primary antibody VE-cadherin (1:50, ab15106; Abcam, Cambridge, United Kingdom), phalloidin-iFluor 488 (1:150, ab176753; Abcam), and claudin-5 (1:100, ab15106; Abcam) to an Eppendorf tube and incubated for 2 hours in a flat box at room temperature. Thereafter, goat anti-rabbit (074-1506; KPL, Milford, MA) antibody was added to the cells, which were incubated at room temperature for 50 minutes in the dark. Finally, 4',6-diamidino-2-phenylindole dihydrochloride (DAPI) (Sigma) was added to stain the nuclei. The staining was observed under a fluorescent inverted microscope (IX51, OLYMPUS). The nucleus stained with DAPI appeared blue under ultraviolet excitation. VE-cadherin and claudin-5 appeared red, and the whole ghost pen cyclic peptide appeared green.

### RNA Extraction and Real-Time Quantitative PCR

The total RNA was extracted from HUVECs using an RNA extraction kit (RN07; Aidlab, Beijing China), according to the manufacturer's instructions. This was then reverse transcribed into cDNA using the Thermo Scientific RevertAid (K1622; Thermo Fisher Scientific, Waltham, MA), according to the kit's instructions. Real-time quantitative PCR was performed to detect the expression of monocyte chemoattractant protein-1 (MCP-1), intercellular adhesion molecule-1 (ICAM-1), and vascular cell adhesion molecule-1 (VCAM-1) using UltraSYBR (CW0957; CWBIO, Beijing, China). Glyceraldehyde-3-phosphate dehydrogenase was used as an internal control. The relative expression of mRNA was calculated using the 2−ΔΔCt method. The primers of the target gene were synthesized by Wuhan Hycell Biotechnology. The primer sequences are shown in Table 1.

**TABLE 1.**
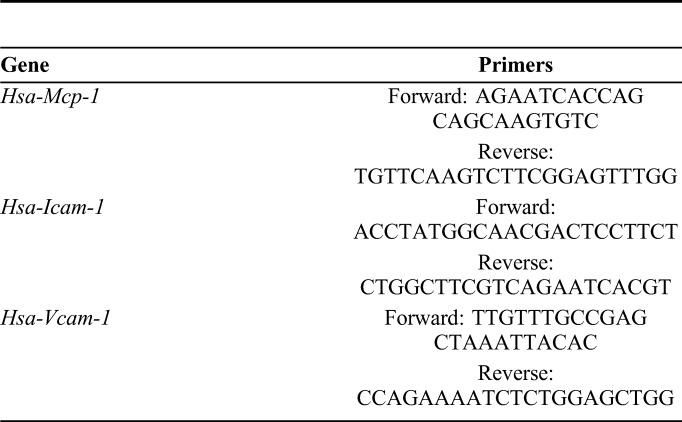
Primers Used for Real-Time Quantitative PCR Assay

### Western Blot

According to the above grouping of cells and mice, the cells or aorta were lysed using RIPA lysate (Beyotime) before centrifuging at 12,000 rpm for 30 minutes at 4°C. The resulting supernatant was used to measure the protein concentration with a bicinchoninic acid kit (Beyotime). The buffers were mixed and heated at 100°C for 5 minutes. The proteins were electrophoresed using a 10% separation gel and a 5% concentrated gel, which were then transferred to a polyvinylidene difluoride membrane (0.45 µm) (Millipore BioRad, Penzberg, Germany) and blocked with 5% skim milk at room temperature for 2 hours. The membrane was incubated with primary antibody SIRT1 (1:1000, 60303-1; Proteintech, Wuhan, China) and β-actin (1:3000, BM0627, BOSTER, Wuhan, China) at 4°C overnight. The membrane was then incubated with goat anti-mouse (074-1506; KPL) at room temperature for 2 hours. An Electro-Chemi-Luminescence kit (Advansta, Menlo Park, CA) was used to detect chemiluminescence on the membrane. The resulting bands were quantified using Image J (Version 6.0).

### Statistical Analysis

SPSS (25.0) (IL) was performed for all data analysis. Images were obtained using GraphPad Prism 5.0 (GraphPad Software, CA). The comparison of the measurement between the different groups was conducted using the one-way analysis of variance. Counting data were represented by frequency using the χ^2^ test and Fisher exact probability method. All experiments were performed at least in triplicate. A *P* value <0.05 was considered to be statistically significant.

## RESULTS

### AD Formation

**FIGURE 1.**
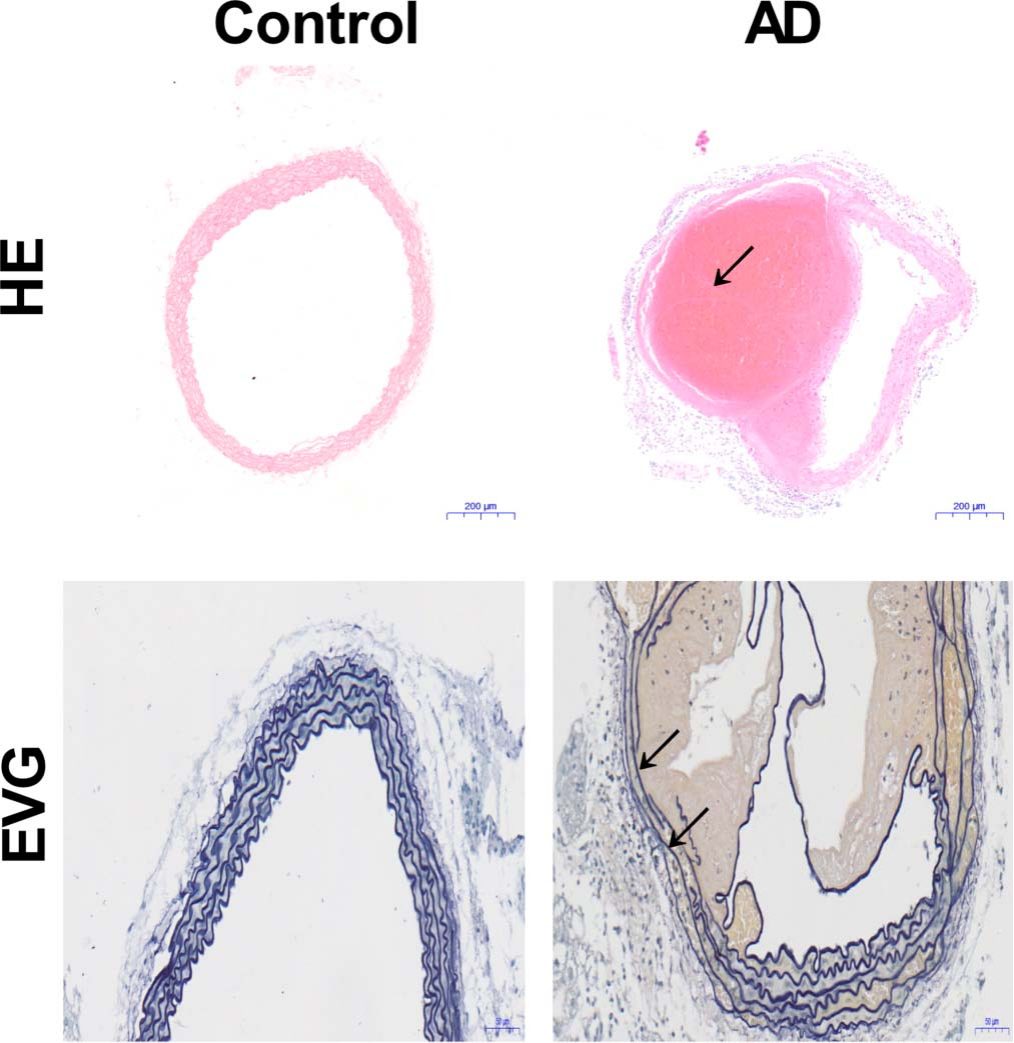
Changes in the aortic structure of mice (n = 20). Although the structure of the aorta in the control mice is complete, the elastic fibers in mice with AD are broken and an aortic pseudocavity is formed (black arrow). HE, hematoxylin and eosin; EVG, Verhoeff–Van Gieson.

The aortic wall of the control mice was intact, whereas the aortic cavity of the mice with AD was composed of true and false cavities. Similarly, while the elastic fibers were intact in the mice without AD, the elastic fibers in mice with AD were visibly broken (Fig. 1).

### RSV Reduces AD and Is Suppressed by EX-527

To study the effect of RSV on AD in mice, we dissected the aorta of the different groups of mice. It was found that the AD of mice was strongly inhibited by RSV because only 30% (6 of the 20) of the AD + RSV group developed an AD, while 75% (15 of the 20) of the AD + Vehicle group developed an AD. These results indicate that RSV greatly reduces the incidence of AD. However, this effect was inhibited by a specific inhibitor of RSV, namely EX-527, because 65% (13 of the 20) of the mice in the AD + RSV + EX-527 group also developed an AD (Table 2).

**TABLE 2.**
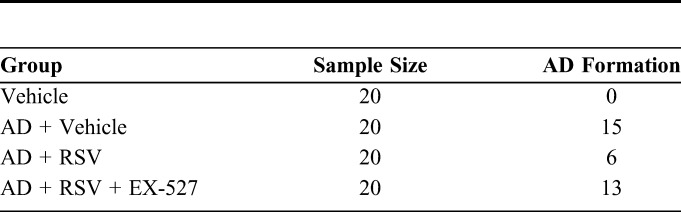
The Number of AD Formation in Different Groups

### RSV Reduces THP-1 Macrophages and LPS-Induced Endothelial Cell Adhesion and Reconstructs the LPS-Induced Endothelial Cytoskeleton to Support the Endothelial Barrier

**FIGURE 2.**
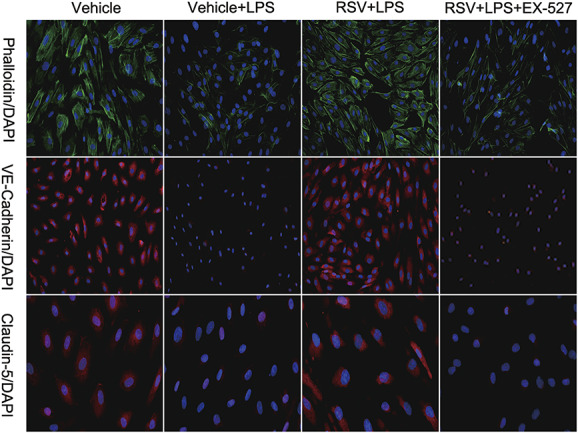
Effect of RSV on the structure and function of LPS-induced endothelial cells (n = 3). RSV improves the LPS-induced structural destruction of endothelial cells. EX-527 eliminates this effect.

**FIGURE 3.**
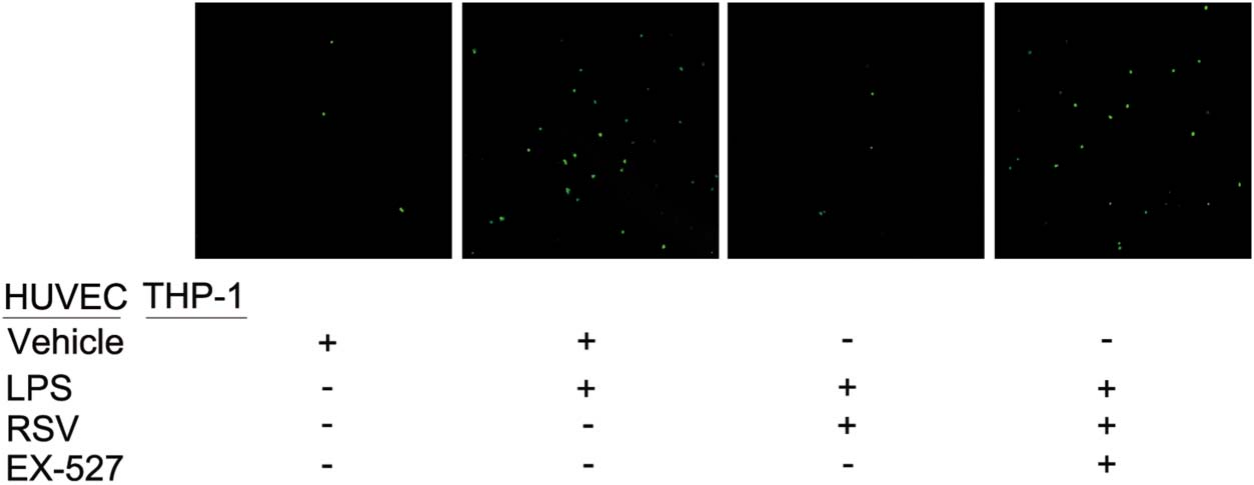
Effect of RSV on the adhesion of LPS-induced endothelial cells to inflammatory cells (n = 3). Fluorescently labeled THP-1 macrophages adhere to LPS-induced HUVECs. Preincubation of RSV with HUVECs can significantly reduce the number of THP-1 macrophage adhesions. EX-527 eliminates this effect.

To study the effect of RSV on the production of chemotactic inflammatory cells by endothelial cells, we investigated the effect of RSV on the adhesion of THP-1 macrophages to HUVECs induced by LPS. THP-1 macrophages were found to mainly adhere to HUVECs induced by LPS, whereas treatment with RSV was found to reduce the ability of THP-1 macrophages to adhere to LPS-induced HUVECs. Immunofluorescence was then used to observe the effect of RSV on the cytoskeleton of LPS-induced HUVECs. Fluorescein isothiocyanate phalloidin immunofluorescence staining showed that the actin structure was neatly arranged and clearly visible in the control group, whereas the actin structure was disordered and varied in thickness in the LPS group. The cytoskeleton was restored. Immunofluorescence staining showed that the fluorescence intensity of VE-cadherin and claudin-5 decreased after LPS treatment and recovered after RSV treatment. However, the above effects were suppressed by treatment with EX-527 (Figs. 2 and 3).

### RSV Reduces the LPS-Induced Expression of ICAM-1, VCAM-1, and MCP-1 mRNA in Endothelial Cells by Increasing SIRT1 Expression

**FIGURE 4.**
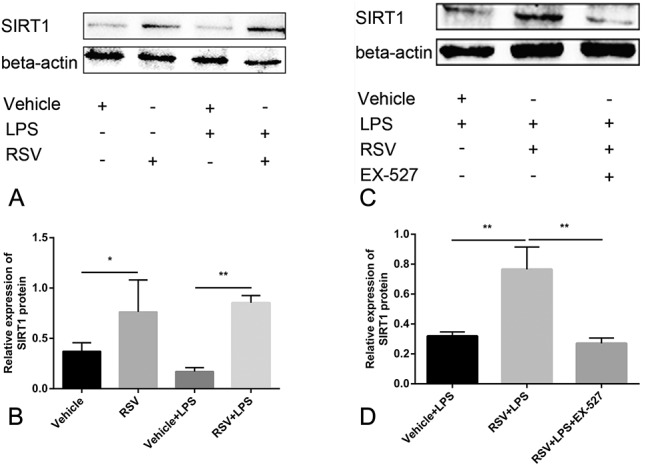
RSV increases the expression of SIRT1 in endothelial cells and is suppressed by EX-527 (n = 3). A, B, Compared with the Vehicle group, RSV significantly increased the expression of SIRT1; the expression of SIRT1 in the RSV + LPS group was significantly higher than that of Vehicle + LPS group. C, D, The expression of SIRT1 in the RSV + LPS + EX-527 group was lower than that of the RSV + LPS group; (A, C) is Western blot, and (B, D) is quantitative. *P < 0.05, **P < 0.01.

**FIGURE 5.**
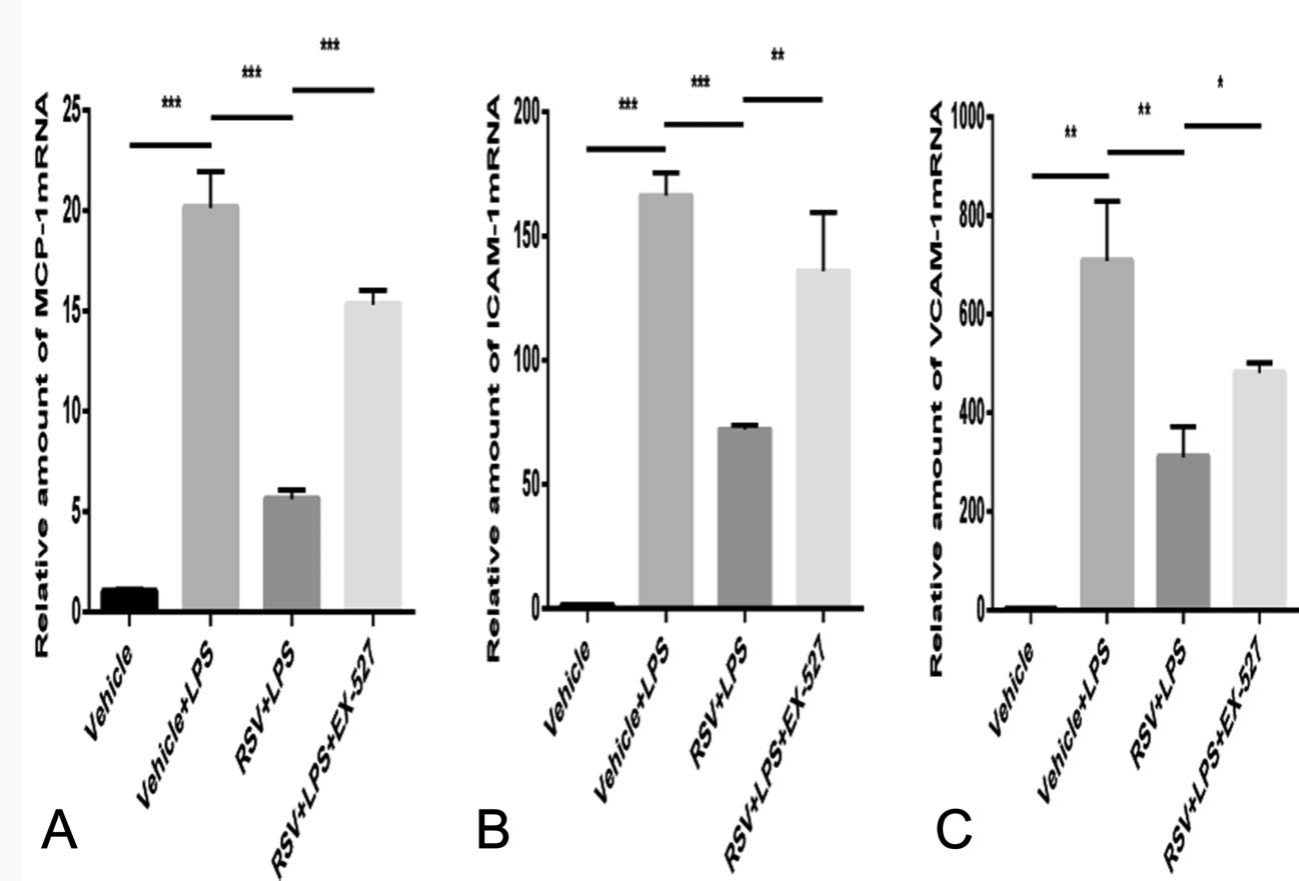
RSV reduces the expression of (A) MCP-1 mRNA, (B) ICAM-1 mRNA, and (C) VCAM-1 mRNA in LPS-induced endothelial cells and is suppressed by EX-527 (n = 3). In the RSV + LPS group, the expression levels of MCP-1 mRNA, ICAM-1 mRNA, and VCAM-1 mRNA were significantly lower than those in the Vehicle + LPS group. The expression levels of MCP-1 mRNA, ICAM-1 mRNA, and VCAM-1mRNA in the RSV+ LPS + EX-527 group were higher than in the RSV + LPS group. *P<0.05, **P<0.01, ***P<0.001.

LPS-induced endothelial cells were treated with RSV to study its effect on the expression of SIRT1, adhesion factors, and chemokine in endothelial cells. We first tested whether RSV increased SIRT1 expression in endothelial cells. As a result, SIRT1 expression was found to be significantly increased in the RSV group. Next, we tested whether RSV was able to reduce the expression of ICAM-1 mRNA, VCAM-1 mRNA, and MCP-1 mRNA in endothelial cells induced by LPS. RSV was found to significantly reduce the levels of ICAM-1 mRNA, VCAM-1 mRNA, and MCP-1 mRNA expression. To confirm that the effect of RSV on endothelial cells was dependent on an increase in SIRT1 expression, we treated the endothelial cells induced by LPS with a SIRT1-specific inhibitor, EX-527. EX-527 was found to eliminate RSV and reduce LPS-induced endothelial cell SIRT1 expression, as well as eliminate the effect of RSV on endothelial cell adhesion factors and chemokines induced by LPS (Figs. 4 and 5).

### RSV Reduces Inflammatory Cell Infiltration in Mice with AD by Increasing SIRT1 Expression

**FIGURE 6.**
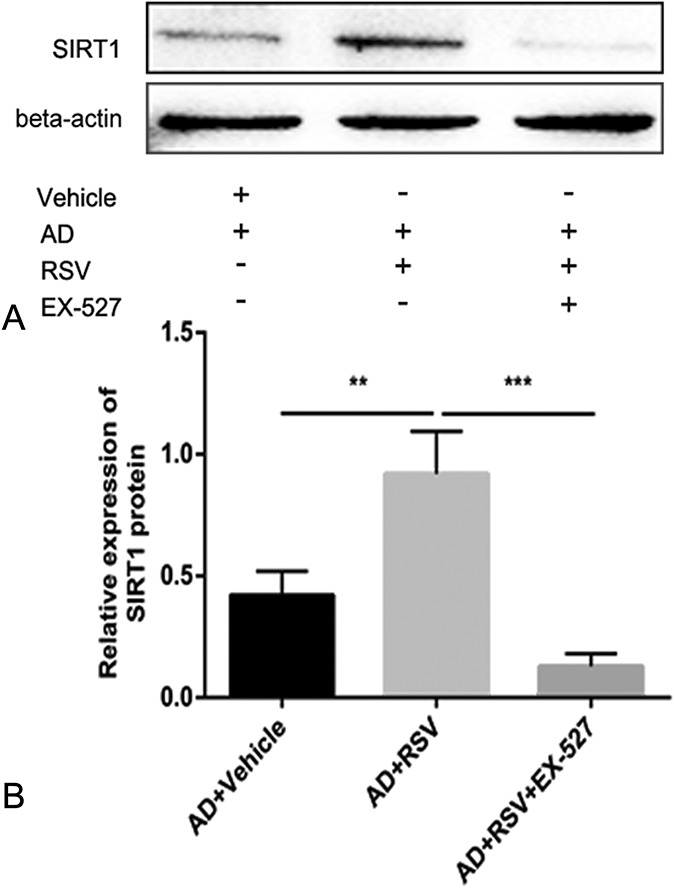
RSV increases the expression of SIRT1 in AD of mice and is suppressed by EX-527 (n = 20 per group). Compared with the AD + Vehicle group, SIRT1 expression in the aorta of mice was significantly increased in the AD + RSV group, while SIRT1 expression in the AD + RSV + EX-527 group was lower than in the AD + RSV group. A is Western blot, and (B) is quantitative. **P < 0.01, ***P < 0.001.

**FIGURE 7.**
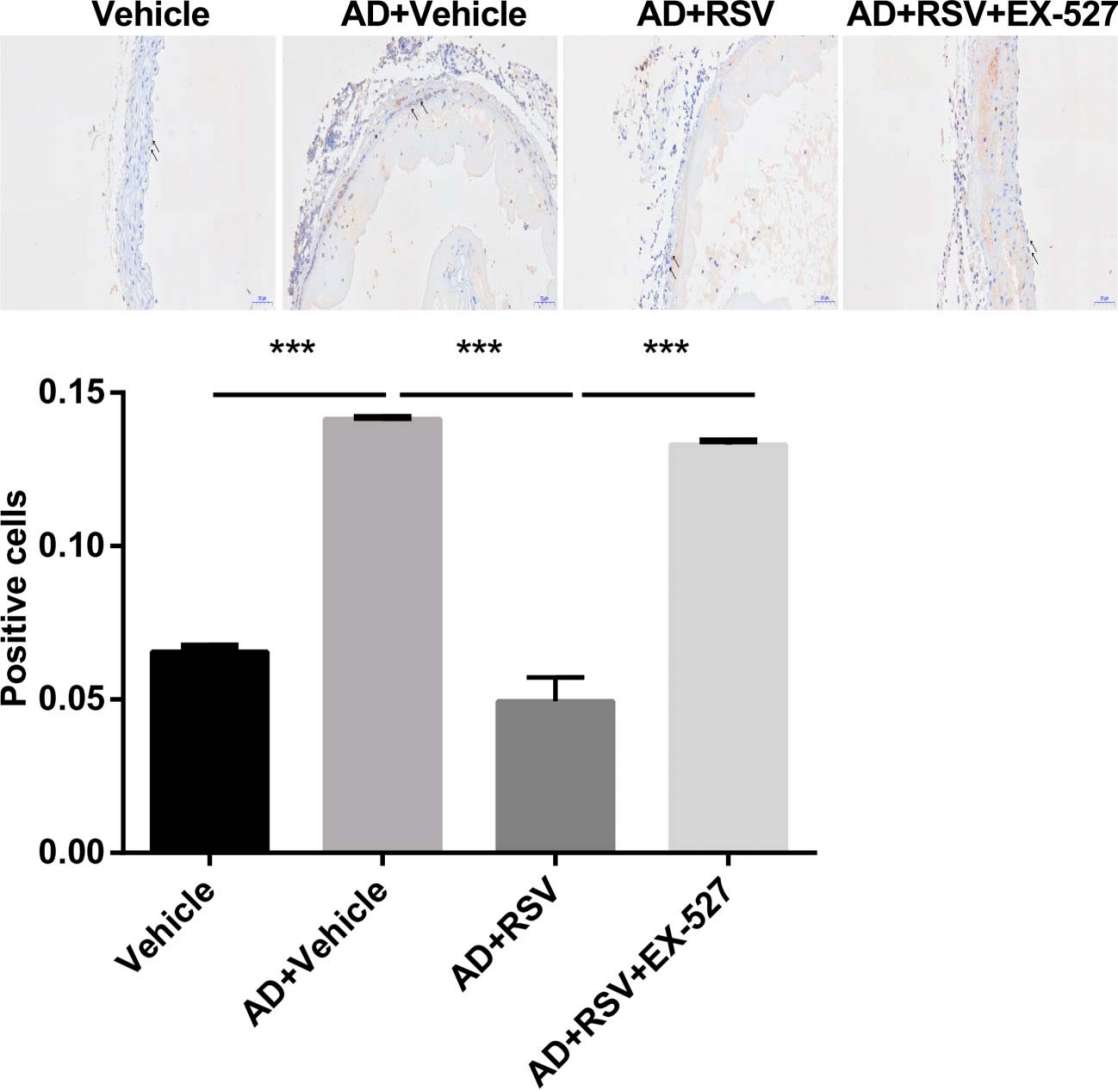
RSV reduces inflammatory cell infiltration in AD of mice and is suppressed by EX-527 (n = 20 per group). Compared with the Vehicle group, a large number of macrophages were expressed in the AD + Vehicle group of mice; the content of macrophages in the AD + RSV group of mice was significantly lower than that in the AD + Vehicle group; the content of macrophages in the AD + RSV group of mice was significantly lower than that in the AD + RSV + EX-527 group. Black arrow represents positive cells; ***P < 0.001.

The inflammatory response plays an important role in AD. RSV is an SIRT1 activator and is able to suppress the inflammatory response. As such, we measured the expression of SIRT1 in the aorta of mice and compared the levels of macrophages (CD68+). The results showed that compared with the AD + Vehicle group, the expression of SIRT1 in the aorta of the AD + RSV group was significantly higher, which is consistent with our results of SIRT1 expression in the HUVECs induced by LPS in the RSV-treated mice. On the other hand, the mice in the AD + RSV group had significantly reduced levels of macrophages, indicating that the inflammation response in the aortic wall of the mice treated with RSV was reduced. To confirm that the effect of RSV on mouse AD was dependent on increased SIRT1 expression, we treated BAPN-induced AD mice with an RSV and SIRT1-specific inhibitor, EX-527. EX-527 was found to eliminate the effects of RSV, namely the increased SIRT1 expression and reduced macrophage levels. These results correlate with the effect of RSV inhibition in mice with AD, namely the increase in SIRT1 expression (Figs. 6 and 7).

## DISCUSSION

The mechanism involved in the AD animal model is mainly the loss of aortic structural integrity, which is characterized by middle-layer cystic necrosis, disordered elastic fiber arrangement, and rupture.^1^ The cross-linking of elastic fibers and collagen fibers is the key to maintaining the integrity of the middle layer of the aorta. BAPN is a lysyl oxidase inhibitor that can inhibit the cross-linking of elastic fibers and collagen fibers, thereby inducing the formation of AD.^17^ Moreover, the modeling rate using BAPN is relatively high and the cost is low. Numerous animal models of AD have shown that the inflammatory response contributes to the development of AD, which is in turn regulated by inflammatory cells and inflammatory factors.^1^ RSV is able to limit the progression of aneurysms and has anti-inflammatory effects. Furthermore, aortic aneurysms and ADs share similar pathogeneses. As such, we chose RSV as the treatment in a mouse AD model induced by BAPN to determine the impact of RSV on AD. In this study, we found that RSV was able to prevent the occurrence of AD. More importantly, we found that the protective effect involved an increase in the expression of SIRT1 by endothelial cells, which resulted in a reconstruction of the structure of endothelial cells, as well as a reduction in the recruitment of inflammatory cells by endothelial cells and the inhibition of the inflammation response. As a result, the occurrence of AD was suppressed.

In the mouse model of AD, we found that the weight of the AD mice was significantly lower than that of the normal mice; at the same time, we also found that the aortic wall structure of the normal mice was intact, whereas the aortic lumen of AD mice formed a true cavity and a fake cavity, wherein the elastic fibers were visibly broken. These pathological changes are consistent with the pathological changes observed in AD in humans.^18^

Previous studies have found that endothelial cell barrier dysfunction and inflammatory responses play an important role in AD. During the inflammatory response, LPS can destroy the intercellular connections, weaken the physical barrier effect of endothelial cells, promote the adhesion of inflammatory cells to endothelial cells, and further aggravate the inflammatory response.^19^ Our study found that the endothelial cytoskeleton changed after HUVEC's injury was induced by LPS. By contrast, the endothelial cytoskeleton was restored after RSV treatment. In THP-1 and LPS-induced endothelial cell adhesion experiments, we found that THP-1 macrophages mainly adhered to HUVECs induced by LPS; however, RSV reversed this phenomenon. As such, the effect of RSV on endothelial cells was eliminated by the SIRT1-specific inhibitor EX-527. The effect of RSV on the endothelial cytoskeleton and the ability of THP-1 macrophages to adhere to HUVECs indicate that RSV is able to repair HUVECs' endothelial structural damage induced by LPS and reduce the chemotaxis of inflammatory cells by endothelial cells.

Endothelial cells are induced to secrete MCP-1 by external stimuli. MCP-1 is a chemokine that can effectively recruit monocytes and promote further development of inflammation.^20^ ICAM-1 and VCAM-1 are 2 of the most important and well-studied endothelial cell adhesion molecules and play an important role in the inflammatory response. These 2 adhesion molecules bind to their corresponding ligands, thereby generating a specific immune response.^21^ Specifically, ICAM-1 and VCAM-1 play important roles in the adhesion and migration of monocytes.^22,23^ Our experimental results show that RSV increases the expression of SIRT1 in endothelial cells and reverses the decrease of SIRT1 expression in endothelial cells induced by LPS; by contrast, RSV reduces the expression of MCP-1 mRNA, ICAM-1 mRNA, and VCAM-1 mRNA in HUVECs induced by LPS. RSV exerts its effects on the aortic endothelium, the cellular effect of which can be suppressed by EX-527. Therefore, we believe that in the process of inflammatory response, endothelial cells secrete chemokines and adhesion molecules in inflammatory sites such as MCP-1, ICAM-1 and VCAM-1. RSV attenuates these processes in the inflammatory response by increasing the expression of SIRT1 in the endothelial cells.

It is well known that RSV is an SIRT1 activator and has anti-inflammatory effects.^24^ This study found that macrophages were significantly infiltrated in the AD of mice in the AD + Vehicle group. However, compared with AD + Vehicle group, the levels of macrophages in RSV-treated mice with AD were significantly reduced, and the SIRT1 content in the aorta of RSV-treated mice with AD was significantly increased. Regarding the effect of RSV on LPS-induced HUVECs' injury, RSV was found to increase the levels of SIRT1 expression in endothelial cells and reduce adhesion between THP-1 and LPS-induced endothelial cells. To confirm that the effect of RSV on mouse AD was dependent on SIRT1, we used SIRT1-specific inhibitor EX-527 on mice and endothelial cells, and we were pleasantly surprised to find that RSV had the abovementioned effects in both the mice and the endothelial cells. The protective effect can be eliminated by the SIRT1-specific inhibitor, EX-527. Based on these results, we believe that RSV reconstructs the structure of endothelial cells by increasing the expression of SIRT1, while reducing the recruitment of inflammatory cells by endothelial cells and inhibiting the inflammatory response, thus suppressing the occurrence of AD.

Although this study provides a new strategy for the prevention of AD, further research will be needed to fully understand how RSV affects AD. Furthermore, this study is yet to be applied in human experiments. Although our results indicate that RSV provides protection against AD, this will need to be confirmed by further research and will be the topic of our future research.
